# The importance of tolerating interstices*: Babushka* markets in Ukraine and Eastern Europe and their role in maintaining local food knowledge and diversity

**DOI:** 10.1016/j.heliyon.2020.e03222

**Published:** 2020-01-17

**Authors:** Renata Sõukand, Nataliya Stryamets, Michele Filippo Fontefrancesco, Andrea Pieroni

**Affiliations:** a Università Ca’ Foscari Venezia, Italy; b Università di Scienze Gastronomiche, Pollenzo, Italy

**Keywords:** Informal markets, Local gastronomic knowledge, Local ecological knowledge, Food policy, Space in-between, Food culture, Ukraine, Qualitative research in food marketing, Quality of life, Legislation, Media education, Food economics, Culture heritage, Anthropology, Business

## Abstract

*Babushka* informal markets selling several homemade gastronomic plant and animal-based products and culinary preparations, as well as wild and cultivated plants, and sometimes family butchered barnyard animals are extremely popular in Ukraine. In this field study that we conducted over a few years we inventoried the most relevant food plant products sold in these markets and we analysed how these markets represent remarkable food refugia for several local niche foods.

In addition, we researched the historical and socio-economic reasons for the start, survival, and revival of this phenomenon, which had its origin during the Communist period. We furthermore evaluated similar recent trends in other Eastern European countries and especially those which had a very different post-Communist trajectory with the aim of addressing the possible factors affecting their survival and what could be done to preserve their existence. In particular, in a few of these countries (i.e. Azerbaijan) we observed how informal food markets represent experimental fields where gastronomic knowledge is not only “preserved”, but also reinvented, possibly in response to the preferences and requests of a city's customers.

## 1 Introduction

One of the most peculiar encounters that visitors to many town streets in various countries of the former Soviet Union may have is that with *babushkas*, e.g. grannies selling their own home-grown produce, foraged ingredients and/or homemade foods. In Ukraine they are present at every open-air market, normally sitting and standing at the fringes of formal markets because at formal markets one has to pay for the selling space. Some *babushkas* are quite shy and modest, while others proactive in reassuring buyers of the quality of their products, proud of their goods and their authentic qualities.

This is not only a kind of “heaven” for Western ethnobiologists but also for local “foodies” interested in gastronomic diversity and tastes. The main scientific questions underlying this remarkable phenomenon are mainly three: why are *babushka* markets still so popular in transition economies; what were the circumstances under which they originated; and what are the diverse environmental, socio-political, socio-cultural, juridical, and economic factors that have affected their existence and survival.

Selling on the fringes is an adaptation to formal rules as market authorities do not gladly welcome the *babushkas* in their territories. The main laws currently regulating the markets in Ukraine (“On the basic principles and requirements for safety and quality of food”), which were passed between 1998 and 2017, require that all food now sold in agro-food markets needs to undergo proper analysis (Law, 2017). Such a requirement automatically excludes the *babushkas* from the official market place, as the quantities they sell are usually small and profits are insufficient to finance all required analyses or even to pay for space in the market. Yet, the Lithuanian experience of the changes in farmer certification that came along with entering the EU provides good examples of the creativity of sellers (Harboe Knudsen, 2010; Mincyte, 2009), although such practices are highly susceptible to corruption, which has already been documented with respect to such markets in Ukraine (Round et al., 2010). Thus, the perceived security brings about a new risk (cf. Blaikie et al., 2014) —the accelerated loss of traditional ecological knowledge.

We argue that along with following food safety regulations, there is a need for the creation of a formal space for supporting, on a limited scale, the informal sale of self-produced food. This research report provides an overview of past and present situations of *babushka* markets in Ukraine, as well as discusses the reasons for the popularity of the sale of home-made foods and compares this information with some other countries where recent changes have occurred. This work will contribute to the ongoing discussion regarding the importance of alternative food networks (AFN), emphasizing a short supply chain, high quality and environmental friendly production methods (see Renting et al., 2003 for full definition of AFN) and the ways in which to sustain traditional food production.

## 2 Data and methods

**Figure 1:**
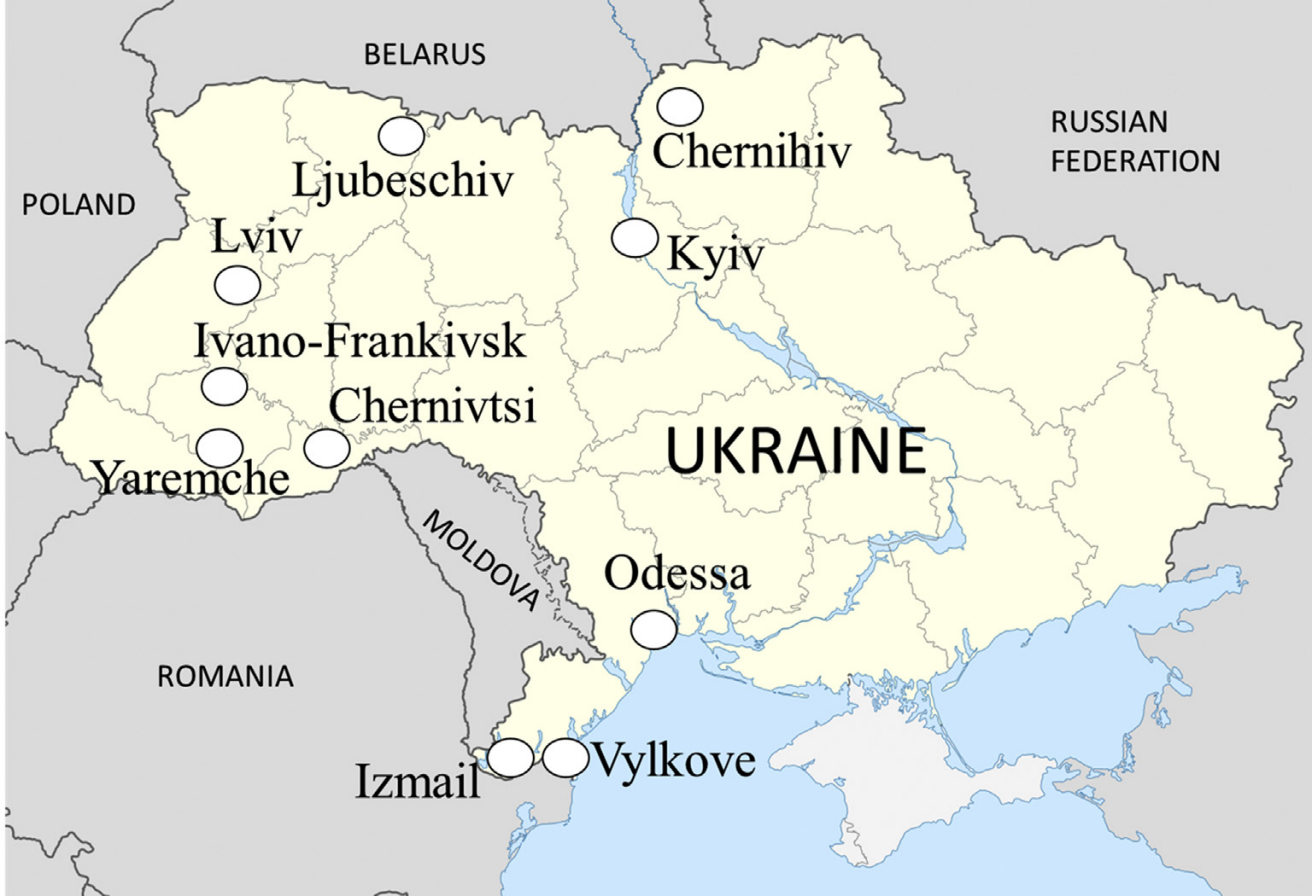
Map of the Ukrainian cities, towns and villages where *babushka* markets were visited. Source of the map: https://commons.wikimedia.org/wiki/File:Ukraine_location_map.svg.

In our discussion we rely on personal observations of various types of markets visited during 2015–2019; the authors made 28 visits to 20 different markets in ten localities across Ukraine (Table 1, Figure 1). Numerous informal conversations were conducted during those visits, often during the causal purchase of goods sold in the market as well as with people buying from the *babushkas*. The market visits took place in the framework of our ethnobotanical fieldwork in rural Ukraine from the same period, when many of the home food-preservation practices were observed during the documentation of the use of wild food plants (Pieroni and Soukand, 2018, 2017; Sõukand and Pieroni, 2016 and forthcoming publications). The field observations and informal conversations are contextualized with respect to the literature. Parallels were drawn on the basis of the authors’ experience and field observations in several towns and cities in other post-Communist countries (Estonia: 1990–2017; Azerbaijan: 2017–2018; Albania: 2004–2018; and Romania: 2010–2015). All the research was conducted in public space and no personal data recorded by any means on the people observed or those with whom informal conversations were held. The ethical guidelines of the Society of Ethnobiology (ISE, 2006) were rigorously and routinely followed.

**Table 1:** Visited markets in Ukraine.

Place	Name of the market	Date visited	Description of the market
Chernivtsi	Нижній ринок/Nizhnіj rinok	28–29.05.2015	Central market of a small town
Chernivtsi	Букоϑинський ринок/Bukovins'kij rinok	29.05.2015	Peripheral market of a small town
Ivano-Frankivsk	Старий ринок/Starij rinok	29.05.2016	Central market of a small town
Yaremche	Ринок/Rynok	28.05.2016	Souvenir market on the road
Ivano-Frankivsk	Ринок "Центральний"/Rynok "Central"nyj"	26, 29–30.05.2016	Central market of a small town
Chernihiv	Рынок/Rynok	20.10.2016	Central market of a small town
Ljubeschiv	Рынок/Rynok	25.10.2016	Small market in a larger village
Kyiv	Вирли⃛я/Vyrlycya	30.06.2016, 12.01.2017	Medium-size market at the metro station in a major city
Kyiv	Лесной ринок/Lesnoj rynok	19.10.2016 and 29.10.2016	Large market at the metro station housing many local small-scale producers
Kyiv	Житний ринок/Zhytnyj rynok	29–30.10.2016	Large market at the town centre housing local small-scale producers
Kyiv	Бессарабский ринок/Bessarabskyj rynok	29.10.2016	Large market at the town centre oriented toward tourists
Odessa	Приϑоз/Pryvoz	8.01.2017 and 10.01.2017	Central market near the main train station
Odessa	Ноϑый базар/Novyj bazar	9.01.2017	Central market near the main local station
Odessa	Сеϑерный рынок/Severnyj rynok	9.01.2017	Town market in the “bedroom suburb”
Vylkove	Рынок/Rynok	5.01.2017	Small market in a larger village
Izmail	Рынок/Rynok	6.01.2017	Central market of a small town
Lviv	Кракіϑський ринок/Krakivskyi rynok	14.06.2018, 22.02.2019–3.03.2019	Large town market
Lviv	Приϑокзальний ринок/Pryvokzalnuy runok	22.02.2019–3.03.2019	Market near the train station
Novoiavorivsk	Ринок, базар/Rynok	22.02.2019	Small local market
Ivano-Frankove	Ринок, базар/Rynok	25.02.2019	Small local market

## 3 Results and discussion

### 3.1 What are the *babushka* markets and how did they originate

Selling household surplus agricultural and wild foods in markets has had its roots in the Soviet and post-Soviet realm. In the Soviet system, the agricultural sector was part of a planned economy in which rural household members had to contribute to the workforce of state and collective farms, similar to that of factories in cities, which broke the “traditional autonomy of smallholders” (Gudeman and Hann, 2015: 9). In the 1950s, Soviet peasants were obligated to supply a portion of their products to the state at a very low price, but as compensation they were allowed to sell their surplus products on the free market, which offered a much higher price due to great demand; a large proportion of the urban food supply was derived in this manner (Nove, 1953). An American visiting the Soviet Union in 1955 described food availability in Russia as follows:

“The bread is relatively cheap and any employed person can afford to buy any amount of bread that he desires, particularly if he is willing to eat rye bread. Most of other food items, such as fresh milk, meat and fruits and vegetables were not available in the state food stores I visited. However, these items were available in the free or collective farm markets at prices substantially above the state store prices. ... The large share of food intake comes from the private plots on most farms”.

(Johnson, 1956).

The Soviet peasant personal household co-existed with collective institutions, utilizing resources of collective and state farms in order to supplement self-provisioning. In the 1960s about 50% of cows belonged to the private sector in Ukraine (Newth, 1961), and by 1965 subsidiary plots of the families of collective farm workers covered five million hectares, while state farm and urban workers used another 3.2 million hectares (Ostrovskiy, 1988, 33, cited by Wegren, 2014). Yet, as articulated by Sätre and Asztalos Morell, (2014 : 266): “citizenship was defined in a way based on work. If you did not work, you did not contribute to the building of socialism”. Hence in the Soviet Union every adult had to have formal employment and only retirement and special circumstances excused someone from contributing to the workforce. The household economy was managed alongside paid labour, during free time. Much of home-production was labour-intensive, ineffective and time-consuming. Yet home production was unofficially substituted by collective facilities to motivate their low-paid workers. For example, young livestock could be bought very cheap, crops which would otherwise have been lost due to ineffective agricultural practices were later picked by village inhabitants, and machinery support was provided privately in exchange for alcohol or other goods; and even the bartering of collective farm goods (grain, gasoline, etc.) was common (see also Rumer, 1981; Small, 2007; Visser et al., 2015 for comparison).

The quality of consumer goods improved greatly in the last decades of socialism (Gudeman and Hann, 2015: 10); however, throughout the former Soviet republics, the 1980s and 1990s are remembered as a time when there was a lot of money, but you could not buy much for that money, as there was very little diversity and a limited availability of products (cf. Round et al., 2010). Market prices were often very high, as there were no products available elsewhere. Therefore, products had more value than money and a lot of goods changed hands in barter exchanges, and thus many networks were developed to secure such exchanges. Hence, the majority of people, not only rural inhabitants but also city-dwellers, grew their own food at dachas/summerhouses or on small pieces of land within the city provided by the municipality.

In the post-Soviet period, along with the triumph of the capitalism (Aage, 2005), economic uncertainty increased and money became more valuable than goods. The whole economic system started to change in order to decrease the role of the state in the production and consumption of goods. Privatization and the return of lands, animals and property to the people created a solid foundation for intensifying the marketing of home-made products. To draw a parallel with Estonia: cows were widely kept (one or two in each household) during Soviet times, and the milk was collected centrally as it was profitable to sell it to collectors; in fact during the earlier years of the Soviet Union it was an obligation. After collective farms were closed in the 1990s, cows and other animals that were once collectivized (see Girnius, 1988 as an example of the process in Lithuania) were returned to the people and an informal market economy (see also Davis, 1997 for a similar de-collectivization process in Latvia and Lithuania) flourished, and suddenly almost everyone became an entrepreneur or farmer. However, this ended quite quickly (in part because governmental level small-scale agriculture was not supported) and market-oriented domestic food production ceased to exist, again confirming the idea of Freidmann that labour intensive small-scale agriculture cannot exist without the support of market capitalism (Friedmann, 1980). During this period it was unacceptable to be poor (cf. Sätre and Asztalos Morell, 2014), so people tried to find another way to prosperity and as a result exchanged unsustainable rural households for city life.

Hence, Ukrainian *babushkas* represent in some sense the remains of the Soviet system of the legal sale of homemade products and are dinosaurs or transitory survivors, which are doomed to extinction in the regulated environment relying on industrially produced food.

### 3.2 Uncommon gastronomic biodiversity sold in markets and why it remains so popular

**Figure 2:**
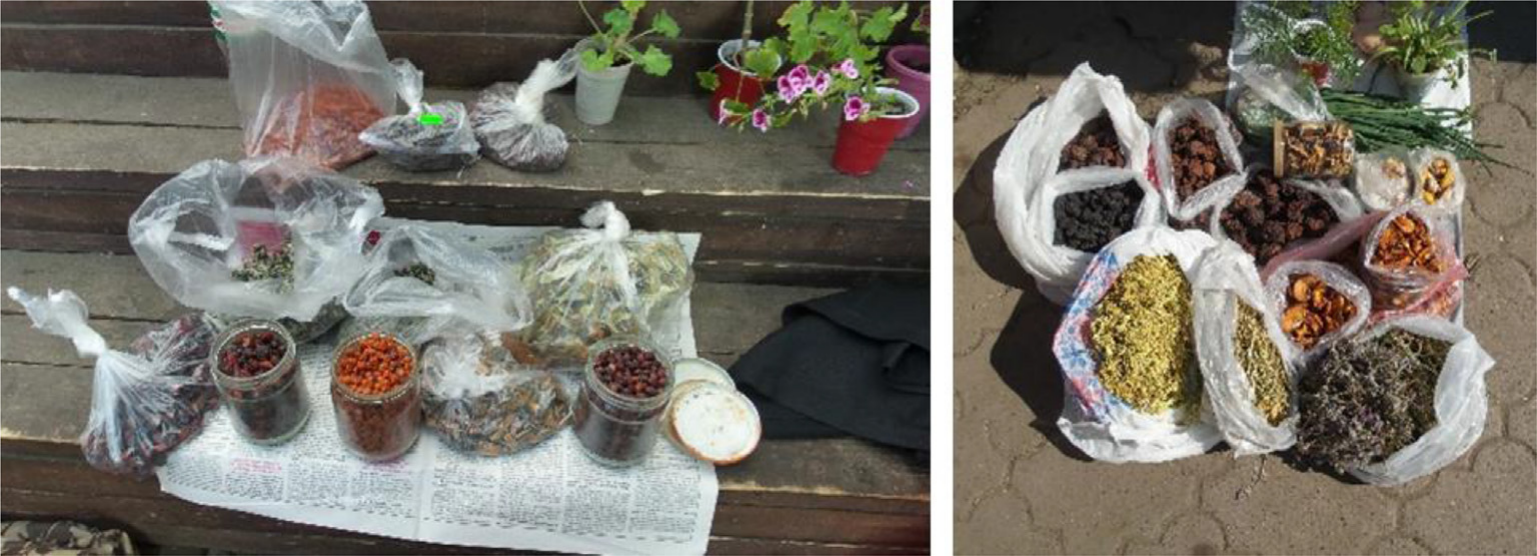
*Babushkas'* dried herbs sold on the fringe of the Ivano-Frankivsk Central market on 28.05.2016.

**Figure 3:**
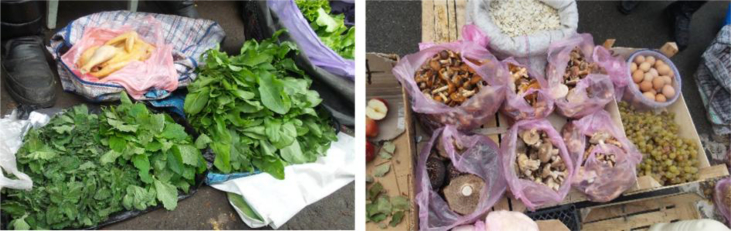
Examples of wild vegetables and chicken in Chernivtsi market, 27.05.2015, and mushrooms and pumpkin seeds in Lesnoj market, Kyiv, 29.10.2016.

**Figure 4:**
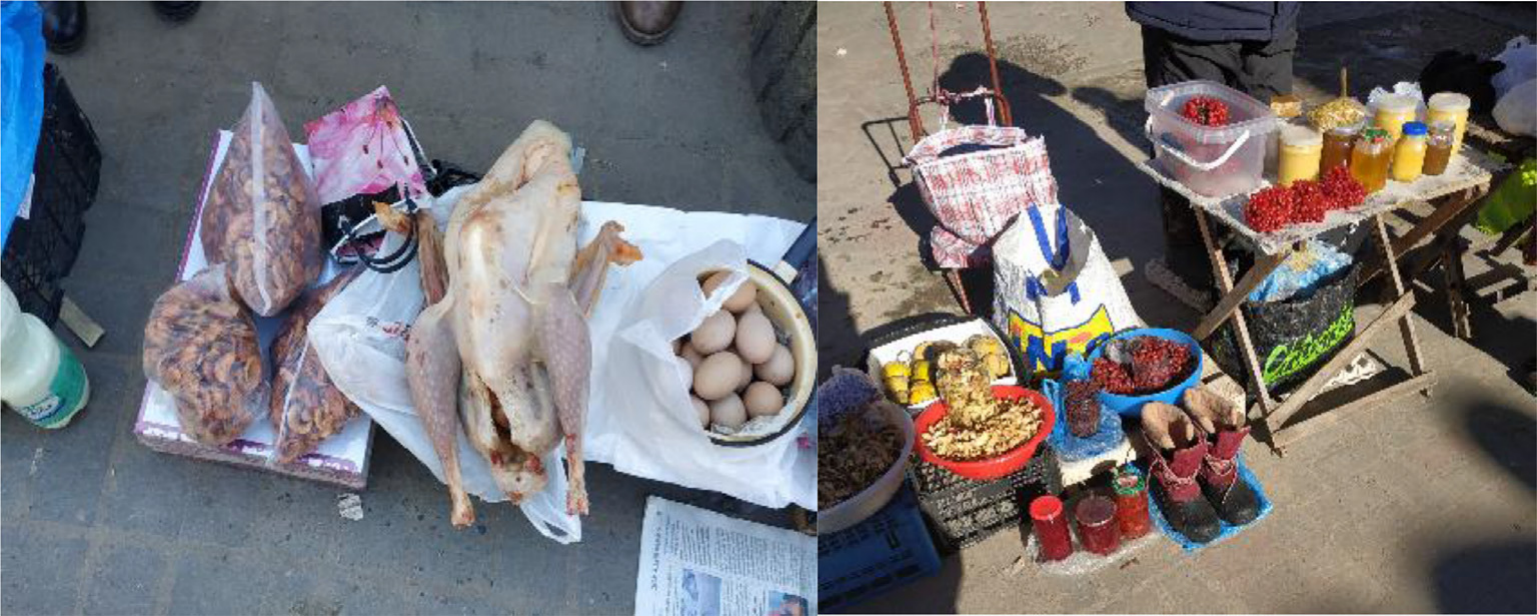
Local products in Lviv markets: milk, eggs, turkey and dried apples (on the left), and *lecho*, apples, guelder rose *Viburnum opulus*, dog-rose fruits *Rosa* spp., quince *Cydonia oblonga* and a variety of honey and bee products (on the right). Visited on 22.02.2019.

**Figure 5:**
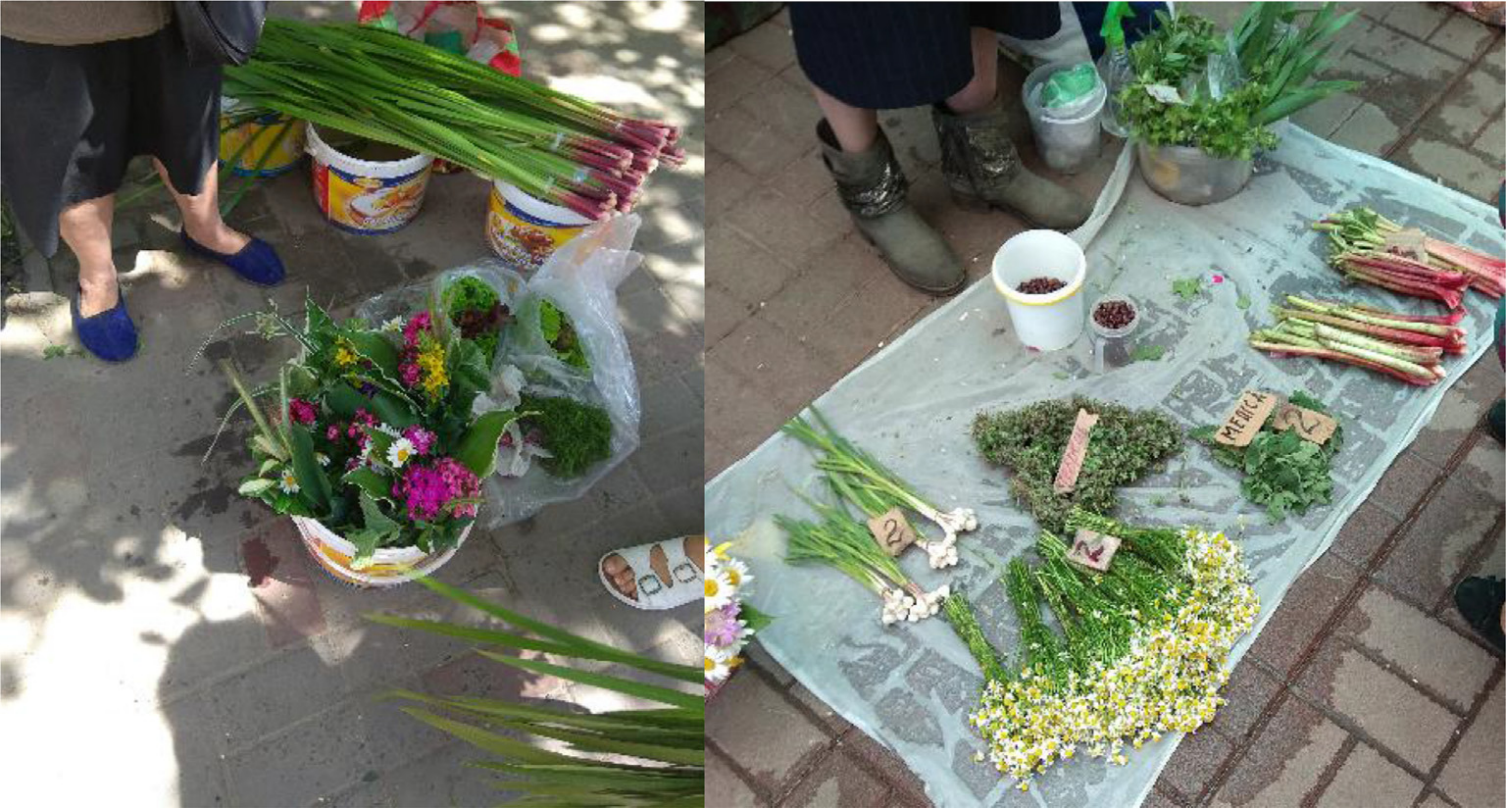
Local products in Lviv markets: wild plant bouquets, calamus *Acorus calamus* and home-grow salad (on the left), and beans, *Thymus* spp., lemon balm *Melissa officinalis*, rhubarb and garlic (on the right). Visited on 14.06.2018.

**Figure 6:**
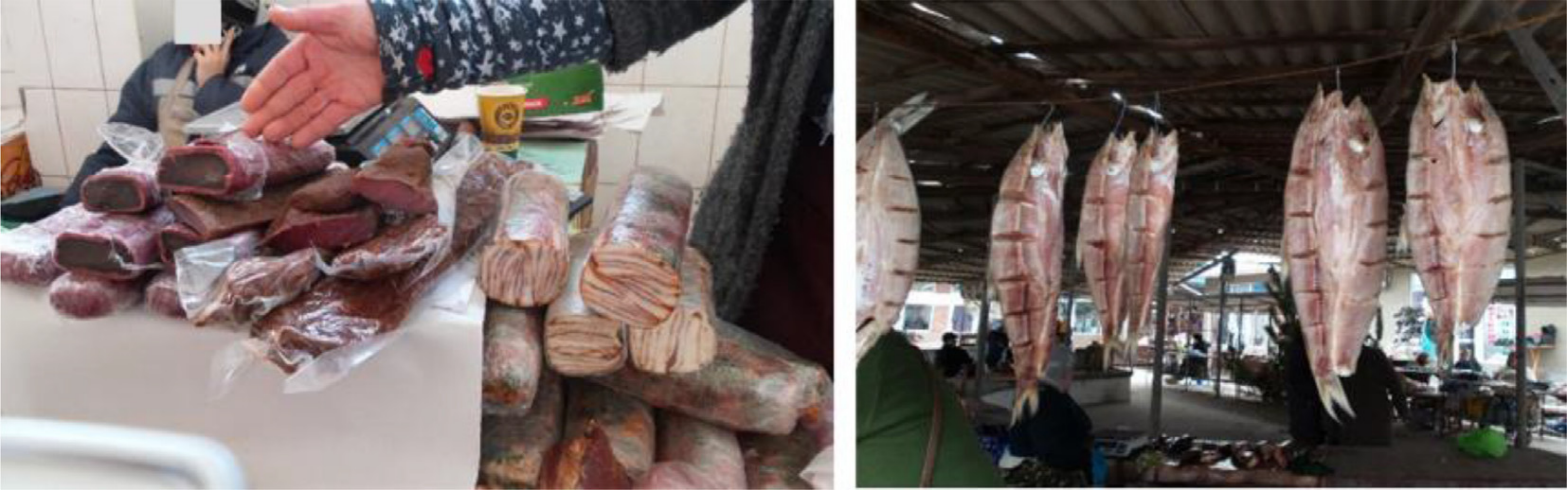
Examples of artisanal cured meat in Odessa Privoz market, 10.01.2017, and dried fish in Vilkovo market, 05.01.2017.

**Figure 7:**
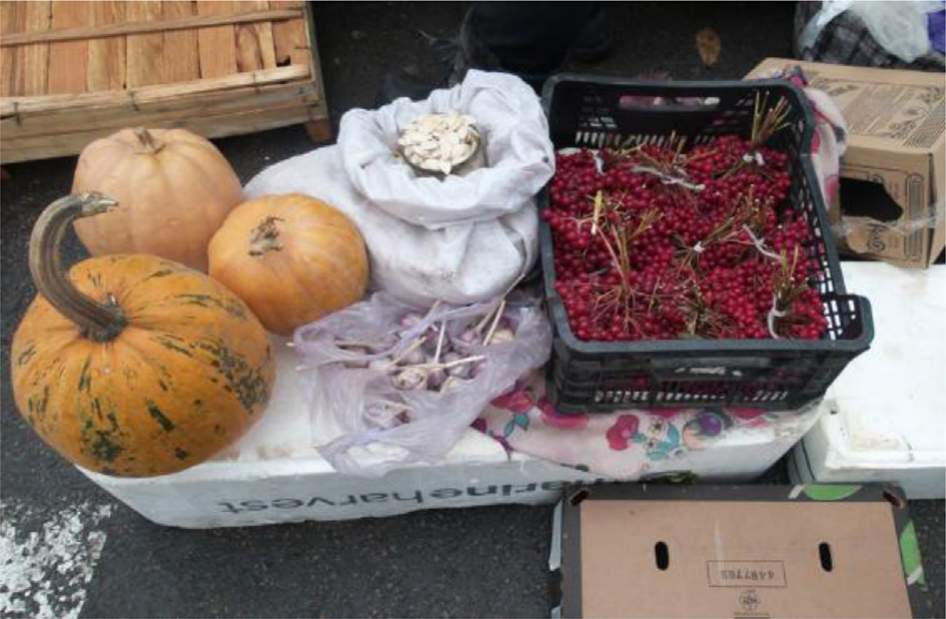
Fruits of *Viburnum opulus* (on the right) sold in Lesnoj market, Kyiv, 29.10.2016, together with home-grown pumpkins and garlic.

**Figure 8:**
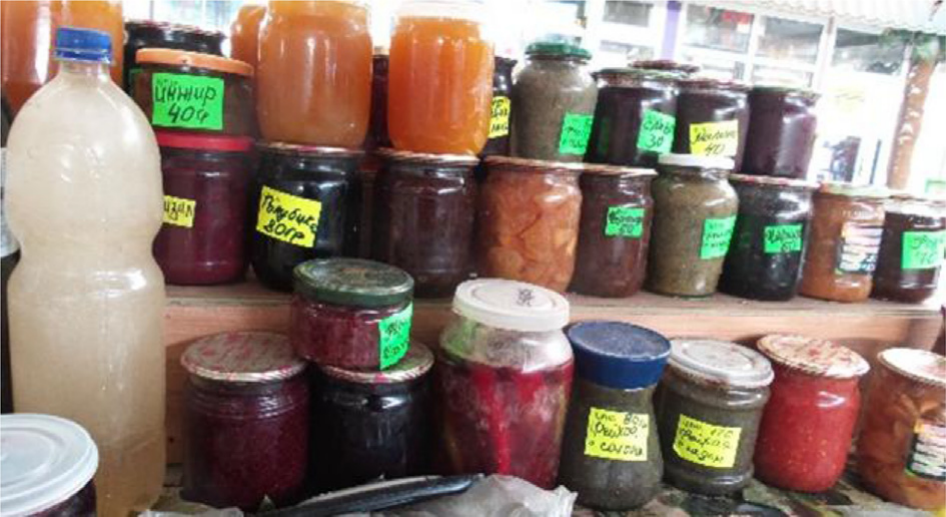
A variety of vegetables and fruit conserves sold in Odessa Novyj market, 09.01.2017.

We documented a wide variety of home-made and artisan food products sold in the *babushka* markets (Table 2). The products were sold both in the official market place and outside the formal boundaries of the market (Figures 2, 3, 4, 5, 6, 7, and 8). Table 3 summarizes the most important factors affecting the popularity of home-made food sold in the markets from the both seller's and customer's perspective.

**Table 2:** Home-made and unusual artisan products sold in visited Ukrainian markets and frequency of their occurrence.

Name and type of the product	Local name	Descriptor	K	IF	Ch	Od	Lv	SM	Comments
**Cured meat and fish**									
horse salami	махан (колбаса из конины)	A	x			xx			includes horse fat
cured horse meat	казылык, баструмаа (вяленая конина)	A				xx			several varieties with different methods and spices used
dried salted fish	сушеная рыба	A	xx			xx	xx	xx	several taxa of fish
smoked fish	копченая рыба	A	xx			xx	xx	xx	several taxa of fish
smoked pork	ветчина, шинка	A	xx	xx	xx	xx	xx	xx	wide variety of products
cured and/or smoked veal, beef	копчена телятина	A	xx	xx	xx	xx	xx	xx	also with different species
salted pork	сало	A/S	xx	x	x	xx	xx	x	also with different species (garlic, chili pepper, black pepper, etc.)
smoked salo	копчене сало	A					xx		smoked pork fat (often marinated in herbs)
homemade salted fat	сало	S	xx	x	x	x	x	x	sold in pieces (fresh) or preserved in 1–3 L jars.
homemade pork conserves	тушонка з свинини	S	xx	xx	xx	xx	xx	x	sometimes sold from under-the-counter; often sold as leftovers of slaughtered pig, of which meat is eaten, but fat left as unneeded
blood sausage with buckwheat	кровянка	S			x		x		viscera filled with blood and buckwheat, pre-boiled
white sausages with buckwheat	ковбаски	S			x		x		viscera filled with boiled buckwheat and fatty meat, pre-baked
blood sausages	кровянка	S			x		x		viscera filled with blood and cereals, pre-baked
blood tripe with giblets	кровянка	A				xx	x		blood and giblets are baked in tripe, ready to eat
**Dairy products**									
curd made from fresh milk	сир, творог, мягкий сир	S/A	xx	xx	xx	xx	xx	x	wide variety of products
curd made from stewed milk	творог из топленого молока	S/A				xx			part of the curd was dark, like coloured with chocolate
cow milk	молоко	S/A	xx	xx	xx	xx	xx	xx	sold in reused plastic bottles or bottles brought from home
goat milk	козье молоко, козяче молоко	S/A	x			x	xx		sold in reused plastic bottles or bottles brought from home
colostrum pudding	молозиво	S/A	xx			xx			seasonal, in winter sold even on streets in Kyiv
dill cheese	сир з укропом	S				xx	x		regional, home-made
cumin cheese	сир з кмином	S	xx	xx					regional, home-made
hard cheese	твердий сир, плавленний сир	A					x		home-made cheese with eggs and milk (sold mostly in summer time)
fresh/sour cream	сливки/сметана, вершки, солодка сметана	S/A	xx	xx	xx	xx	xx	xx	with a very high fat content so that “spoon stands in it”
sour milk	кисляк, кисле молоко, ряженка, ґуслінка	S/A	xx	xx	xx	xx	х	x	wide variety of products with different preparation methods
*brynza*	бринза	S/A	xx	xx	xx	xx	хх	x	mainly home-made, could be smoked, with different seasonings, hard, soft or small particles
butter	масло	A/S	xx	xx	xx	xx	х	xx	mainly artisan, only few sold as surplus; seasonal selling in summer time
**Fresh plants and mushrooms**									
fresh sorrel *Rumex acetosa*	щавель, кислец	W/C	xx	xx	xx		xx		seasonal
fresh red *Atriplex hortensis*	лебеда, лoбеда	C		xx					seasonal, common in the Ivano-Frankivsk market
raw edible mushrooms	гриби	W	xx	xx	xx		xx	xx	seasonal, many species sold widely at markets or on the sides of the road
unripe fir cones	шишкі хвої	W		x			x		seasonal, sold for jam and medicine
fir shoots	лісточки хвої	W		x	x		x		seasonal, sold for making jam
horseradish *Armoracia rusticana*	хрін, хрен	W	xx	xx	xx		xx		fresh root, seasonal
sea buckthorn *Hippophae rhamnoides* fruits	облепиха, обліпиха	C	x				x		seasonal
guelder rose *Viburnum opulus* fruits	калина	W	xx			xx	x	xx	seasonal
cultivated garlic	чеснок, часник	C	x			x	xx		seasonal
wild garlic *Allium ursinum*	ведмежа цибуля, черемша	W					xx		sold in early spring
strawberries *Fragaria vesca*	суниця, ягоди	W		xx	xx		xx	x	seasonal
blackberris *Rubus fruticosus*	ожина						xx		seasonal
raspberries *Rubus idaeus*	малина						xx		seasonal, wild and home-grown
cranberries *Vaccinium oxycoccos*	клюква, журавина	W	xx			xx	xx	X	sold also in winter
cowberries *Vaccinium vitis-idea*	брусниця	W	xx				xx		seasonal
pseudo-acacia *Robinia pseudoacacia* flowers	акація	W			x				sold for making jam
**Dried plants and mushrooms**									
dried mushrooms, mostly *Boletus edulis, Leccinum* spp.	сушені гриби	S				xx	xx	xx	sold by chain or by kilo
dried wild pears and apples	сушка	W	x	x		x	xx	x	sold in mixture and separately
dried sorrel *Rumex acetosa*	щавель	S				x	x	x	packed in sachets
rowan *Sorbus aucuparia* fruits	рябина, горобина	S		x			x		for making recreational tea
dog-rose *Rosa* spp. fruits	шипшина	S	x			x	x		for making recreational tea
hawthorn *Crataegus* spp. fruits	боярышник, глід	S	x			x	x		for making recreational tea and medicine
wide variety of dried herbaceous plants for making recreational and medicinal teas	травки	A	x		x	x	xx	x	specialised *babushkas*, approached by clients as specialists and asked for their advice concerning health issues; they collect some or even most of the plants they sell themselves; knowledge mainly book-derived or referred to books as a source of authority
**Pickled/salted plants and mushrooms**									
fermented/boiled/salted sorrel *Rumex acetosa*	щавель, кислец	S/A	xx	xx	xx	xx	xx	x	sold in 0.5 L jars
pickled cucumbers	солені/квашені/мариновані огірки	S/A	xx	xx	xx	xx	xx	x	in jars and loose; wide variety of added ingredients, including wild taxa
pickled tomatoes, red and green	солені/квашені/мариновані помідори	S/A	xx	xx	xx	xx	xx	x	in jars and loose; wide variety of added ingredients, including wild taxa
pickled cabbage	квашеная капуста, квашена капуста	S/A	xx	x	x	xx	xx	x	sold by weight, cut and whole, wide variety of tastes and seasonings
pickled plums	квашеные сливы	A	xx						fermented with salt and sugar, sold by weight
pickled paprika	квашеный перец	A	xx						fermented with salt, sold by weight
pickled apples	квашеные яблоки	A	x			xx			fermented with salt and sugar, sold by weight
pickled watermelon	квашеный арбуз	A	x			xx			fermented with salt and pumpkin pulp, sold whole
pickled melon	квашеная дыня	A				x			sold whole
pickled eggplant	квашеный баклажан	A	x						fermented with salt, sold by weight
salted/marinated mushrooms	солоні/мариновані ґриби	S	xx	xx	xx	xx	xx	xx	sold in 0.5 or 1 L jars, mostly marinated
preserved horseradish (with red beet)	хрен (с буряком), бурячки з хроном	S/A	xx	x	x		xx		stored in jars for short-term preservation, for Easter and Christmas holidays
*Lecho*	лечо	S	xx	x	x	x	xx	x	home-made preserves, highly variable content, sold along with other preserves
*Adzhika*	аджика	A/S				xx	xx		sold in small quantities with short preservation period (fresh) and long preservation period (boiled)
**“Unusual” jams and compotes**									
sea buckthorn *Hippophae rhamnoides* jam	варение из облепихи	S	x	x		x	x		home-made
guelder rose *Viburnum opulus* jam, compote	варение из калины	S	x			x	x	x	home-made
raspberry jam/compote	Варенье/компот из малины	S	x	x	x	x	x		home-made
rose *Rosa* spp. petal jam	варенье из розы	S	x	x			x		home-made
bog bilberry *Vaccinium uliginosum* jam	вареніе з голубики	S	x	x	x	x	x		home-made
mixed fruit compotes	ягідні компоти	S	x	x	x	x	x	x	wide variety of products
**Others**									
veal maw	телячий желудок	S				x	x		seller's reaction to our question was quite unfriendly: “this is meant for those who know what it is without asking” – she probably did not want to explain, as there are just a few people who know what to do with it
fat of goose	гусиный жир	S				x			sold in small jars mainly for medicinal purposes
fat of goat	козий жир	S				x	x		sold in small jars mainly for medicinal purposes
home-made pancakes	блинчики, млинці, налисники	S				x	x		lady selling pancakes was surrounded by a crowd, many of whom had been waiting her arrival; various fillings
home-made curd cakes	творожная запеканка	S				xx			made from leftovers not sold in previous days
home-made cakes	домашня випічка	S				x	x		different kinds and types of cakes, sold mostly before important holidays
boiled wheat	Кутя	S	x				x		used in Christmas dishes, probably boiled in greater amounts and surplus sold in the street-markets of large cities (Kyiv, Lviv)
*Varenyku*	вареники з картоплі і сиру, солодкі з чорниці, вишні	А					xx		home-made and ready to eat, sold close to tram stations and where people leave from work and want to eat home-made food but have no time to prepare it themselves
bouquets for pickling	набір для квашення та маринування	W/C					хх		horseradish roots and leaves, dill seeds, oak leaves, leaves of black currant and cherries for pickling cucumbers and tomatoes, etc.

Abbreviations: Sellers in the market: x –1-3 sellers at a location, xx – more than 3 sellers at a location; Descriptor: S – Surplus/food made for market by private person, A – Artisan, W - collected from wild, C – Cultivated; Markets: K – Kyiv, IF – Ivano-Frankivsk, Ch – Chernivtsi, Od – Odessa, Lv – Lviv; SM – Small markets.

*Adzhika* – ground tomato base with garlic, chili pepper, carrots, nuts and many different ingredients and seasonings; *Brynza -* cheese made from cow, sheep, or goat milk or a mixture of milk with a special ferment*; Lecho* – ground tomato base with bell pepper, carrots, onion and other varieties of fruits and vegetables (apple, chili pepper, zucchini, etc.); *Varenyku* - dumplings with a variety of fillings, e.g. boiled potatoes, cheese or sweet with blueberries, cherries, etc.

**Table 3:** The factors affecting the popularity of home-made food sold in markets.

Factors/Side	Sellers	Customers
*Economic*	Optimizing the use of domestic surplus and earning some cash to support families	Saving money allocated for acquiring food ingredients
		Exclusive home-made, time-consuming products
*Social*	Possibility to continue rural activities and lifestyle; face-to-face interactions with customers	Face-to-face interactions with producers
		Responsibility for helping the rural inhabitants
*Health-related*	Indirect improvement via an increase in income	Home-made food products perceived as healthier than industrial or large-scale agricultural ones
*Cultural*	Recognition of the value of small scale agriculture and artisanal, domestic work	Nostalgia for village life, childhood tastes, “authenticity”, old recipes, “grandma's” food

From the perspective of the customer home-made food sold in the market holds various meanings. It bears symbolic value as it is produced locally and it provides an opportunity to interact with sellers. Sellers, on the other hand, have the opportunity to achieve/increase financial sustainability and thus continue practicing a more or less traditional/accustomed lifestyle and sustainably using resources (getting rid of surplus products or leftovers), but also interact with (returning) customers. Customers can actually influence and shape what sellers offer by requesting a few forgotten preparations (nostalgic revival of foods that recall memories) and also sometimes novel food products (creation of new “traditions”). In the visited markets, for example, most wild vegetables, old landraces of cultivated crops, and animals products belonged to the revival food group, while the fruits of *Viburnum opulus*, widely sold in autumn, to the new “traditions” (cf. Pieroni and Soukand, 2018).

From an economic perspective the sale of home-made food can be viewed as a form of response to economic marginalization and a way in which to combat poverty, along with informal work, social networking, gift-giving and mutual exchange involving about 20% of the population (Round et al., 2010). Such practices were also common during Soviet times and represented an important means of survival during the fall of the Soviet Union, particularly following the serious drop in GDP in the 1990s. Unlike in Baltic countries, Ukraine's economic marginalization continued throughout the post-Soviet era: a few years ago (2014–2015) economic growth, after hovering around zero for a while, dropped suddenly and sharply, almost hitting -10%; but then it rose again to 1% in autumn 2016, and at the present time it is showing signs of stabilizing; however, the stability of growth depends on the implementation of comprehensive fiscal and structural reforms (World Bank, 2016). In modern-day Ukraine food expenses consume a large proportion of income, even for the wealthier portion of the population (Makeev and Kharchenko, 1999). The economic rationale of domestic food production is well studied in the academic literature, especially through the quantitative lens (Round et al., 2010 and references therein).

The sale of home-made products in present-day Ukraine should not be perceived only as a means of survival of an economically marginalized people. Mutuality in exchange is also seen as a connection with others as opposed to personal self-interest, implying a degree of empathy, the ability to see yourself through the eyes of others (Gudeman and Hann, 2015: 2). Given the deep historical roots of such a practice, it can be seen as a dialogue between seller and customer and often on a very personal level. It helps maintain contact between the city and the village and creates a situation in which both sides (food supplier and consumer) are eager to communicate. For example, in the outdoor part of Privoz market in Odessa in January 2017, with the temperature around -10 °C, the authors observed the arrival of an older woman with freshly baked pancakes with jam and curd cheese fillings. She lively described her products to 5–6 other women who instantly surrounded her, while distributing some pre-packed products to customers who seemed to have pre-ordered a specific amount. The conversation of the women sounded as though the interaction between seller and buyers had been repeated numerous times and that this time the seller was a bit late due to unexpectedly cold and snowy weather conditions.

It is still widely believed in Ukraine that home-made, and especially domestically grown food, tastes better and is much healthier than that of industrial production. This belief originated during Soviet times, when industrial food had little diversity and was of low quality, and people had to make all the preserves for winter on their own (cf. Round et al., 2010, p. 1208). Similar trends were also observed by the authors in 2013–2017 in modern Belarus, where the growing of one's own food is still important for survival, as mean salary is quite meagre, leaving little resources for purchasing commercial food.

### 3.3 Social values of responsible *babushka* market buyers/customers

Buyers mentioned several reasons for buying from *babushkas*. The main reason was fresh home-made and home-growth products at a very good price, followed by the recognition that in buying those products one was helping rural residents. The buyers also highlighted several categories of products which could be found in *babushka* markets.

The first category is rare products not often found in supermarkets or regular shops. This category includes not only a diversity of forest products, such as fresh berries and mushrooms, birch sap, fresh and dried medicinal plants and tinctures, but also home-made ‘preserves’ based on unique recipes, like rose jam or *lecho*.

The second category is eco products and/or fresh products. People believe that at *babushka* markets you can usually buy fresh products without chemical additives. There is a strong belief that home-growth products do not contain nitrates and other harmful chemicals. Fresh, non-pasteurized milk, cheese and home-made butter were some of the products that could not be found in regular shops, and which taste much better.

The third category is time- and energy-consuming products. Buyers at the *babushka* markets explained that another important issue was the large amount of time needed to prepare specific products like fermented cabbage, home-made butter and cheese, *varenyky* or homemade sausages, and preserves. Some buyers explained that they do not have the option of spending half the day collecting blueberries or mushrooms in the forest and therefore they prefer to buy them from *babushkas*.

Social or responsible buying was highlighted along with cheaper prices and the possibility of communicating and negotiating with the seller. We observed the importance of social relations between buyers and the seller, when the latter has regular customers with whom they have a trusting relationship.

The safety-net function of *babushka* markets is undervalued. The sellers explained that in villages there were no job opportunities and selling their products was the only way to earn cash. Working-age individuals with children that are more than 3 years old are the most vulnerable village dwellers, who have to find a means for survival. They adapt to societal changes by either going abroad or to big cities to earn money, or they have a garden and a variety of animals for providing food and they collect a variety of forest berries and mushrooms (Stryamets, 2016), the surplus of which is sold in markets to generate cash. According to our informants, pensioners and mothers with babies up to 3 years old have a state-supported pension, which often is the only stable income for the whole family (Pantyley, 2009; Stryamets, 2016). The diversity of products sold is impressive, from forest berries, mushrooms, and medicinal plants to a variety of home-made dairy products to live chickens, rabbits, ducks and geese. Often the prices are very low—the driving force behind this is to earn at least a little something. During one of our visits to the market in Lviv, we bought a bouquet of *Helichrysum arenarium* for 3 UAH, which corresponds to about 0.1 Euro, a very small amount of money; e.g. to buy one of the cheapest loaves of bread or to get from the village to the city using public transportation, a person needs to sell 5 bouquets.

The food security function of village dwellers is also underestimated, as they provide products to sustain their own lives, as well as the lives of people living in the city.

As a result, in the local media there are claims that association with the EU will lead to the prohibition of selling dairy, meat and home-growth products at *babushkas* markets, as the health regulations of the EU are stricter than those of Ukraine. Those claims have created a number of myths and legends among rural dwellers, such as the idea that it would be forbidden to sell home-made and home-growth products (Marcinovskyi, 2018). In the 2014 amendments to the Law of Ukraine “On quality and safety of food products and food raw materials” there are control requirements, at laboratories based in markets, for home foods sold at markets.

The first generations of urban dwellers perceive home-made food brought back to town after visiting parents still living in countryside as a symbol of belonging (see for comparison Smollett, 1989 for a discussion on similar practices in Bulgaria). As demonstrated in Lithuania, official warnings about food safety may have limited impact on customers, as perceived good qualities like authenticity and naturalness of the products outweigh the warnings (Harboe Knudsen, 2010). In modern Estonia, which like Lithuania underwent similar processes to those of Ukraine until the 1990s (see Bardone, 2013 for more details on preservation practices), people earning an average salary can easily afford to buy high-quality canned industrial food, and thus, while preserves at home are still sometimes made, the idea of the healthiness of home-made food has been transferred to everyday cooking.

While this may not always be the case, sellers often describe the sale of products as getting rid of by-products of domestic food production in a good and sustainable way. An elderly woman, who was selling one non-standard piece of about 1 kg of salted home-made pork at the informal selling spot outside of Vyrlytsa market in Kyiv in January 2017, willingly described her product as high quality and explained its origin. She claimed that, after killing the pig, children took the meat home, leaving her with much of the pork fat, as it is not an appreciated product. So she decided to salt it, as has always been done, and bring it for sale. The piece represented the last of several kilos she had brought along.

Selling side-products is often perceived as an opportunity to maintain an accustomed lifestyle, as keeping animals also requires some resources. Sayings such as “I can buy a kilo of sauerkraut/bottle of beer from the shop. Who will I make a big brew for?” were often heard by the authors during fieldwork. In Ukrainian villages domestic animals (like cows, pigs, sheep, goats, rabbits) and birds (chicken, ducks, hen, gooses) were still widely kept and plants preserved for winter (fermented, dried, marinated, cooked, smoked) because people use the products themselves, but also because the surplus products can be sold which provides an additional or in many cases the main source of income for the family. The possibility of selling the products seems to be an added bonus for continuing the practice. *Babushkas* keeping just one-cow or the habit of home-fermenting as a way of storing food for winter may have a good chance of survival, if there is a legal space for selling surplus products.

### 3.4 The social structure of *babushka* markets

This research points out the importance of interstitial spaces in the urban landscape of Ukraine and other former Soviet countries. The practices of the *babushka* markets create a bridge that links formal food markets with the household economy (Gudeman, 2016; Hart, 2010). In fact, *babushka* markets represent an interface between these two distinct spheres of exchanges, as Gudeman, (2001, pp. 5–11) pointed out, endowed with specific practices, necessities, values, knowledge, and worldviews, and allows the encounter between actors who, otherwise, participate in different ways and with distant roles in the serendipitous and disjunctive flows that mark contemporary urban space (Heyman and Campbell, 2009), namely urban consumers and rural or peri-urban producers. In an urban space in which the access and use of public spaces are regulated and limited, the interface develops in locations that could be considered as non-places (Augé, 1992), anonymous spaces which are not usually used as platforms for developing relationships between people. The pavements along the main streets, passageways, and roads outside metro stops, and the other places our research has highlighted, are transformed by the presence of the *babushkas* and their products and become sticky places in slippery spaces, to paraphrase Markusen (1996), or, in other words, points of reference and interaction. The *babushka* markets are places of social and gastronomic encounters. The products presented nowadays in the informal stands on the side of the road are a contemporary expression of the gastronomy developed within the domestic sphere over the course of the Soviet era. Even during the final phase, the household played a central role as a reservoir of gastronomic diversity and a workshop for culinary creativity in the face of a formal market limited in the range of products they offered (Jacobs, 2015; Lakhtikova et al., 2019). This gastronomy, which mostly relied on home gardening and subsistence agriculture in urban and rural areas, has continued in the transition period (Ekström et al., 2003; Seeth et al., 1998); however, it had only a limited impact on the development of the post-Soviet market foodscape, which expanded largely through the introduction of new products from abroad (Menkhaus et al., 2005). Thus, in this context, the *babushka* markets are the gateway for domestic products to get in the market. In so doing, they can be seen as a first form of integration of household economy within the broader market economy. They are a creative frontier through which the urban foodscape is enriched in terms of products and knowledge of ingredients, as well as recipes. At the same time, success with regard to the diffusion and stabilization of such semi-formal markets is fostering a substantial formalization of domestic gastronomy, which is otherwise mainly oral and private. In other words, it is producing a positive commodification of this domestic, food tradition. While Comaroff and Comaroff (2009) mostly pointed out the negative implications of the commercial use of elements of local cultural heritage, in the case of the *babushka* markets, implementation establishes a field of interaction that sees urban consumers and *babushkas* come together. In so doing, the urban consumers' demand for new products and heritage complements the *babushkas’* search for supplementary sources of income for the household and the implicit demand for social recognition. Thus, it is not in the cathedrals of modern gourmandism but rather on the side of the road that the profile of everyday gastronomy of the region develops and expands.

### 3.5 Why is state-supported sale of home-made food important?

Round et al. argue that practices born out of economic marginalization “*cannot be considered a true alternative, as in many cases they do not offer long-term security to the household*” (2010, p. 1202). We agree with this proposition but want to add that regardless of the economic and hygienic reservations, such practices should be supported, rather than prohibited, by legislation at the governmental or communal level because the practice of selling home-made food products may contribute to food security and the preservation of traditions that otherwise may be lost. Twenty five years ago, during and after the collapse of the Soviet Union, the largest drop in GDP (between -12 and -18.5% in the years 1991–1994) occurred (Burawoy, 1996), and over half of the population lived below the poverty line and food accounted for over half of average household expenditure in 1991 (Nello, 1992). Yet, only a few people starved to death during that period indicating that there were other economic practices ensuring survival (Round et al., 2010).

Household food production is complex and ambiguous, as every household has its own recipe for even commonly made food (Monova, 2015). The ability to legally sell surplus food products or develop a small-scale, artisan enterprise could contribute to the survival of both rural small-scale household agriculture, which is more flexible and may help to ensure survival in the times of hardship, and variations in the preparation of food from basic ingredients. Analysing the ability of household production in Russia to fill the void in the wake of a food embargo, Wegren (2014) suggests that households (which in Russia produce about 43% of animal husbandry products) are integral to food sovereignty and “are an underutilized resource that can make greater contributions to national food security” (2014, p. 492). Russian agrarian economist Alexander Chayanov (1888–1939) argued that peasant households react to signals of the market in a very distinct way: an increase of income from selling can, instead of increasing production, lead to a reduction of work hours as soon as needed resources are secured (Chayanov, 1966, see also interpretation by Gudeman and Hann, 2015: 6–7). Yet, the same individual food production on personal plots has been blamed for the collapse of large-scale agriculture and perceived as a kind of “peasant revenge” supporting the breakdown of the Soviet empire (Kitching, 1998). In the wider context, the sale of home-made products is a means in which to sustain traditional food production, ensuring the availability of food that does not require industrial production.

Allowing, on a limited scale, the sale of home-made products within close proximity of production would:

a - preserve and promote local practical knowledge, which when lost will be very difficult to restore when truly needed;
b - keep the knowledge distributed within different layers of society, not concentrating it only in a few spots, and thus promoting food security;
c - create better and more frequent contacts between rural and urban populations.

Globally, and in Ukraine particularly, the urban population is growing, as is the demand on the food supply. The *babushka* markets are still the link from rural to urban, providing a win-win strategy – growing local foods, which often is more sustainable than transporting food hundreds of kilometers, and using unique recipes and supporting rural dwellers with additional income. Globally this link is important in terms of the food supply and food security. Therefore there is a crucial need for more research on locally produced food, recipes, and food culture.

### 3.6 Comparison with other Eastern European countries

**Figure 9:**
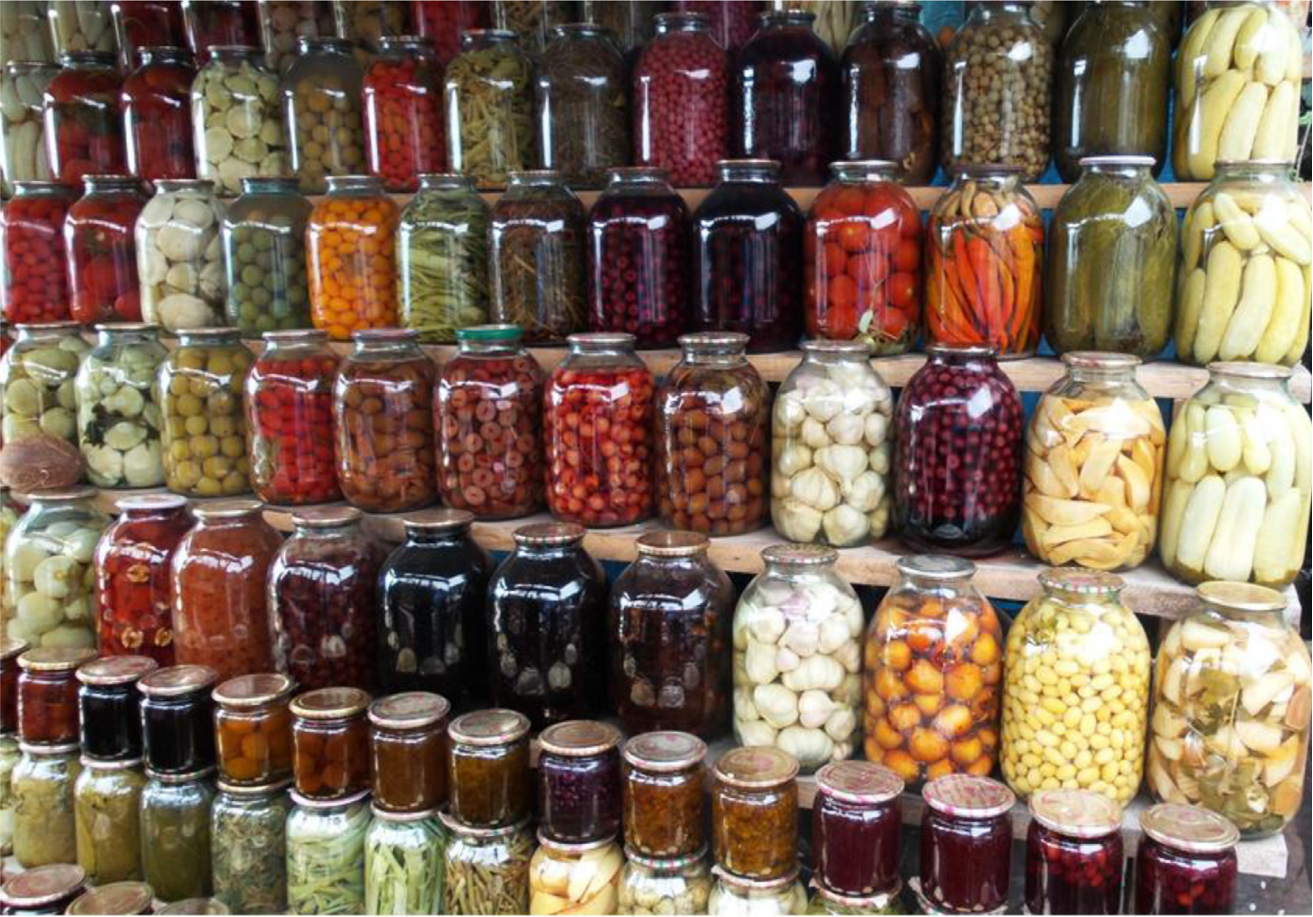
A tiny sample of the home-made fruit and vegetable preserves sold in an informal street market on the main road linking Şhamaxı to Nic, Azerbaijan, April 2018.

Table 4 and Table 5 show the most common wild food plant items sold in the informal markets visited in Azerbaijan (see also Pieroni and Sõukand, 2019, Sõukand and Pieroni, 2019) as well as in Albania, Romania, and Estonia, respectively. The comparison is made only for wild food plants, as this has been the main research focus of the authors to date. The results clearly show that informal markets in Azerbaijan still preserve a remarkable amount of wild plant-based Local Ecological Knowledge and Local Gastronomic Knowledge (Figure 9), compared with other post-communist countries, which instead underwent a huge westernisation process, where mainly only a couple of wild herbal teas and wild or semi-domesticated fruits are still available at informal markets. Some of these informal markets were visited several times over the years and a remarkable decrease of informal spaces, which are normally located at the fringes of bigger markets, was observed.

**Table 4:** Most common wild and semi-domesticated food plant items sold in the informal markets visited in Azerbaijan during the period 2017–2018.

*Allium* spp. (fresh leaves of wild garlic)	
*Asparagus verticillatus* (fresh young wild asparagus shoots)	
*Berberis vulgaris* (fresh, preserved, and brined barberries)	
*Cornus mas* fruits (fresh, dried, and preserved Cornelian cherries)	
*Crataegus* spp. (dried hawthorn leaves, flowers, and fruits)	
*Eleagnus rhamnoides* (fresh and preserved sea buckthorn fruits)	
*Fagus orientalis* (brined Oriental beech leaves)	
*Fragaria vesca* (fresh and preserved wild strawberries)	
*Heracleum* spp. (brined hogweed stalks)	
*Malus baccata* (brined cherry apples)	
*Mespilus germanica* (brined medlar fruits)	
*Prangos ferulacea* (brined young shoots)	
*Prunus cerasifera* (brined cherry-plums)	
*Rumex patientia* and *R. acetosella*. (fresh and brined dock and sorrel leaves)	
*Silybum marianum* (fresh peeled stalks of milk thistle)	
*Viburnum opulus* (guelder rose dried fruits)	
*Viola odorata* (preserved flowers)

**Table 5:** Most common wild food plants sold in the informal markets visited in Estonia, Albania, and Eastern Romania during the period 1995–2018 (items with an asterisk have tended to disappear in recent years.

*Armoracia rusticana* (fresh horseradish root, Romania only)	
Corylus avellana* (fresh and dried hazelnut kernels, mainly in Albania)	
*Cornus mas* (fresh and preserved wild Cornelian cherries, mainly in Albania)	
*Eleagnus rhamnoides* (fresh and preserved sea buckthorn fruits, Romania only)	
*Fragaria vesca* (fresh, in Albania* and Estonia)	
*Hypericum perforatum* (fresh and dried aerial parts of St. John's Wort)	
*Juglans regia* (fresh and preserved unripe fruits and fresh and dried walnut kernels)	
Malus sylvestris* (dried, sliced wild apples)	
*Origanum vulgare* (fresh and dried flowering tops of wild oregano, Albania only)	
*Pyrus* spp.* (dried, halved wild pears)	
*Rumex acatosella* and R. patientia* (fresh and fermented dock and sorrel leaves)	
*Sideritis* spp. (dried flowering aerial parts of wild mountain tea, Albania only)	
*Vaccinium* spp. (fresh or, more rarely, in sweet preserves; *Vaccinium oxycoccos* also preserved in water, Estonia only)

This decreasing trend was in terms of both the number of mainly elderly vendors and products, which are represented nowadays only by wild vegetables, wild fruits, and wild medicinal herbs, and less and less by home-made dairy products, cured meats, and conserves. The reasons for the decline – which was especially evident in Eastern Romania and Estonia – are various:

• restrictions due to the adoption of national food laws, no longer allowing the direct selling of home-made food products by small-scale farmers;
• decrease of surplus in domestic production;
• decrease of the number of small-scale farmers;
• disappearing Traditional Ecological Knowledge or Traditional Gastronomic Knowledge of the gathering/processing/producing of traditional food ingredients and products;
• decrease in demand from urban buyers/consumers in due to the possible perception of the superiority of a farmers' items in terms of genuineness and authenticity.

**Figure 10:**
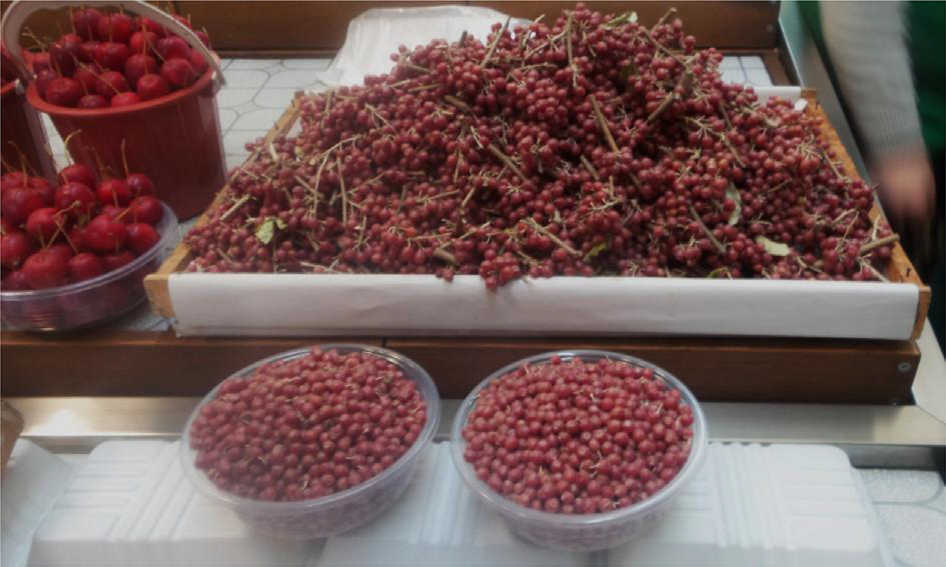
*Malus baccata* and *Eleagnus umbellata* fruits sold in the central Baku markets, November 2017.

It is remarkable that in some Azerbaijani markets farmers found space for introducing “new” food products, such as those based on edible flowers (i.e.*Viola odorata* preserves) or newly introduced cultivated fruit trees native to Eastern Asia or South America (e.g. *Malus baccata*, *Schizandra chinensis* (Figure 10), *Elaeagnus umbellata*, and feijoas *Acca sellowiana*).

### 3.7 Do *babushka* markets have a chance of survival in the near future?

Making food is rooted in the history and culture of a community and requires, depending on the type of food, specific knowledge (what to use), skills (how to recognize, how to make), types of resources (like land and animals) and time. Small-scale home-production of agricultural goods cannot be perceived as an alternative to large-scale agricultural production, yet as a study in post-socialist Russia has shown, this subsidiary practice is superior in that it is socially sustainable, healthy and environmental friendly (Visser et al., 2015). Hence those practices have to be supported as a supplemental activity for ensuring food security, as is common in other EU countries, where a considerable proportion of agricultural production is obtained from small, family-run enterprises. Wegren et al. (2017) show, as a positive example of food sovereignty, that in the European Union programs dedicated to organic farming have been introduced and such production is supported by funding through the Common Agricultural Policy.

Locally produced *babushka* products also give a sense of place and belonging to a specific area, e.g. brynza cheese provides a sense of belonging to mountain communities. Or non-pasteurized milk and homemade cheese evokes memories of childhood spent in the village with grandma. Both farmer and consumer share a common culture and belongingness to a specific region. These benefits provided by *babushka* markets are irreplaceable.

Not all examples are positive, however. In Estonia, official statistics listed nearly 13 000 private households with one or two cows in 2001, while in 2016 there were less than only 750 such households (Eesti Statistika, 2017). Official registration of a cow in modern Estonia is quite complicated for an older person: everything is done electronically and one officially cannot keep a cow not on record, slaughtering of an animal is allowed only in certified slaughterhouses, etc. Tightening of the regulations of production conditions for products sold on the market (like the number of sinks in the kitchen where the food is made, etc.) may drastically affect the enthusiasm and willingness of the people to continue traditional food production beyond one's own family needs. On the other hand, recently relaxed rules for the production and sale of home-made beer in Estonia have resulted in the wide availability of small-scale artisan beers on the market. Paraphrasing the discussion of multiple dimensions of food sovereignty by Iles and Montenegro De Wit (2015), we can say that recognising home-made food as an important cultural phenomenon on the government level may enable people to not only resist marginalization, but also strengthen their sense of identity related to locally grown and processed food.

**Figure 11:**
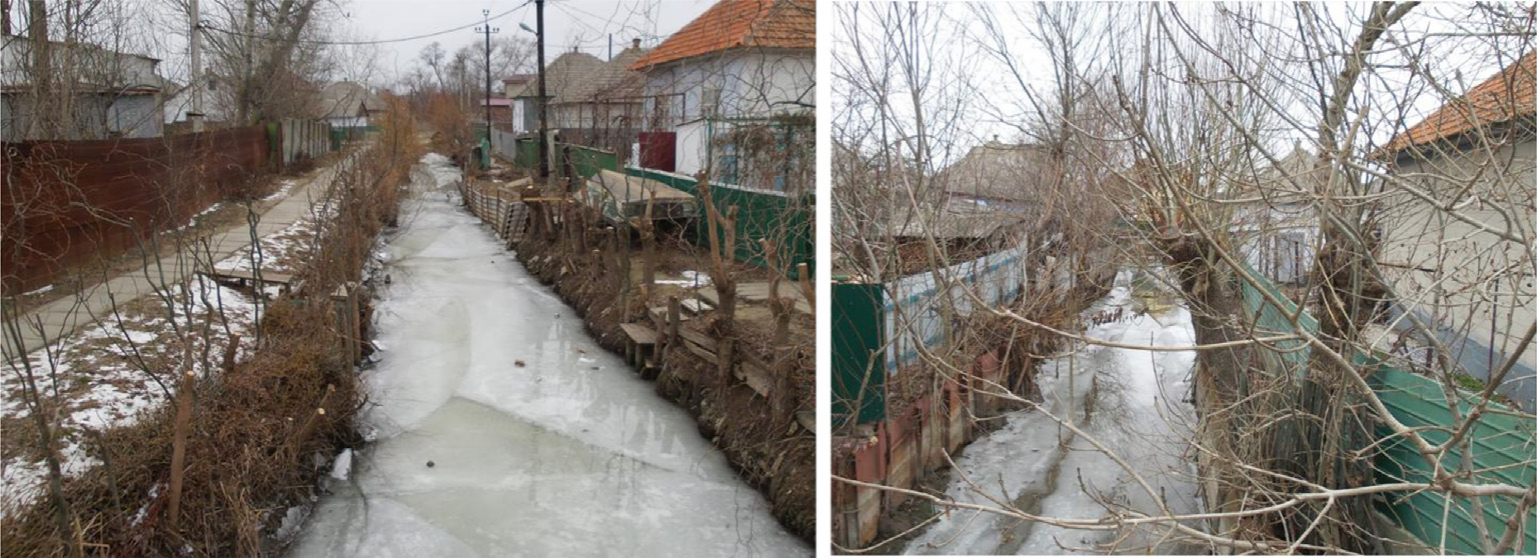
Channels in Vylkovo.

Legislation supporting local food production strategies must be discussed within and approved by local communities. The importance of harmonising legislation with the traditional ways of producing local food and the way it is communicated to people was demonstrated in an informal conversation in Vylkovo, the village of Russian Orthodox Old-Believers famous for their fishing traditions (Van Assche and Teampău, 2015). When we asked about what (wild) plants are collected in the village, one 50-year-old woman selling some products from her home garden in the market responded: “*We've now been relying on what we grow ourselves, otherwise one would not be able to survive. We have no other jobs here. ... Earlier on we were a fishing village, but now only a few of us can afford registration of a boat. Registering a boat is even more difficult and expensive than registering a car. Now everyone can fish only on the small piece of the river going through your land, but look at what it all looks like, no-one takes care of the river any more*” (Figure 11).

As we were buying some apples from her and exchanging few words in English, she realised that we were from Europe and she added, with bitterness in her voice: “*It is all the fault of the legislation imposed on Ukraine by the European Union. Here we have to work hard for you, Europeans, to prosper and live a rich life.*” This rhetoric is typical of how EU policies are often perceived at the fringes of its borders by local farmers, shepherds, and fishermen and how local and regional policies are not able to re-articulate overall super-national frameworks in such a way as to protect the needs of these small-scale actors in the foodscape. In our case study, Vylkovo's locals had practiced fishing for generations and could not adapt independently to new rules overnight.

## 4 Conclusions

Local small-scale home-based food production may not conform to all hygienic and other security standards, yet through being adapted to local ecological and cultural conditions it minimises risks, and should help to preserve traditional ecological knowledge and provides additional security for people. This security fulfils not only basic nutritional needs in times of hardship, but also the social need for human connection through the sharing of food and experience. While designing laws related to local foods, authorities must take into account not only hygiene and product safety requirements, but also the need for sustaining local grassroots food production and consumption, as well as the role home-made products play in not only the economy, but also the social and cultural spheres of life.

Future in-depth studies are needed to evaluate the effects of different regulations and cultural perceptions regarding traditional gastronomic knowledge, beliefs, and skills of producing small-scale food products from basic ingredients, as well as the attitudes of consumers to different possible scenarios of food production. Moreover, urgent field studies are needed to document the traditional use of (especially wild) food ingredients in Ukraine, and to evaluate the diversity within the population of the skills required for domestically preparing food from self-grown or wild basic food ingredients. The vanishing trend of such skills in countries that have already joined the EU or that are on the road to membership attests to the importance of developing alternative educational programs for preserving or revitalizing such skills in Ukraine and beyond, and a more *open-minded* food legislation that leaves space for interstitial and informal distribution channels of gastronomic bio-cultural diversities.

## Declarations

### Author contribution statement

Renata Sõukand: Conceived and designed the experiments; Performed the experiments; Analyzed and interpreted the data; Wrote the paper.

Nataliya Stryamets: Performed the experiments; Analyzed and interpreted the data; Wrote the paper.

Michele Filippo Fontefrancesco: Analyzed and interpreted the data; Wrote the paper.

Andrea Pieroni: Conceived and designed the experiments; Performed the experiments; Analyzed and interpreted the data; Wrote the paper.

### Funding statement

Research conducted since 2018 has been supported by the European Research Council under the European Union's Horizon 2020 research and innovation programme (grant agreement No 714874). Earlier fieldwork was supported by the University of Gastronomic Sciences, the Estonian Science Foundation (Grant IUT22-5), and the European Union through the European Regional Development Fund (Centre of Excellence in Estonian Studies, CEES).

### Competing interest statement

The authors declare no conflict of interest.

### Additional information

No additional information is available for this paper.
